# Supplementary material for: Patterns of Intron Gain and Loss in Fungi
Source: PLoS Biol. 2004 Nov 30;2(12):e422. doi: 10.1371/journal.pbio.0020422 (PMC532390; doi:10.1371/journal.pbio.0020422)
Supplement: Table S1 — Also available at http://genes.mit.edu/NielsenEtAl/. (4.3 MB ZIP). [file pbio.0020422.st001.zip › NielsenEtAl/html/841.html]

AN3026.1.NCU10066.1.MG03511.1.FG07939.1


```
 CLUSTAL W (1.82) Multiple Sequence Alignments - Introns Inserted


Sequence 1: NCU10066.1	1223 aa
Sequence 2: MG03511.1	1220 aa
Sequence 3: FG07939.1	1220 aa
Sequence 4: AN3026.1	1210 aa
Alignment Length: 1233 aa
Number Identitical Residues: 859 aa
Alignment Score (without introns) 37991


MG03511.1 	MQASSGMLTK0FESKSSRAKGIAFHPKR2PWILVSLHSSTIQLWDYRMGTLIDRFEEHDG
NCU10066.1	MQASSGMLTK0FESKSSRAKGIAFHPKR2PWILVSLHSSTIQLWDYRMGTLIDRFEEHDG
FG07939.1 	MQSSPGMLTK0FESKSSRAKGIAFHPKR2PWILVSLHSSTIQLWDYRMGTLIDRFEEHDG
AN3026.1  	------MLTK0FESKSSRAKGIAFHPKR2PWILVSLHSSTIQLWDYRMGTLIDRFEEHDG
          	      **** ***************** *******************************

MG03511.1 	PVRAIDFHKTQ~PLFVSGGDDYKIKVWSYQTRRCLFTLNGHLDYVRTVFFHHELPWIVSA
NCU10066.1	PVRGVDFHKTQ0PLFVSGGDDYKIKVWSLQTRRCLFTLNGHLDYIRTVFFHHELPWIVSA
FG07939.1 	PVRGVDFHKTQ~PLFVSGGDDYKIKVWSYQTRRCLFTLNGHLDYVRTVFFHHELPWILSA
AN3026.1  	PVRGIDFHPTQ~PLFVSGGDDYKIKVWNYQTRRCLFTLNGHLDYVRTVFFHPELPWILSA
          	***.:*** ** ***************. ***************:****** *****:**

MG03511.1 	SDDQTIRIWNWQNRSLI1CTMTGHNHYAMCAQFHPKEDLVVSASLDQSVRVWDI~SGLRK
NCU10066.1	SDDQTIRIWNWQNRSLL~CTMTGHNHYVMCAQFHPKEDLIVSASLDQTVRVWDI1SGLRK
FG07939.1 	SDDQTIRIWNWQNRSLI1CTMTGHNHYAMCAQFHPKEDLVVSASLDQSVRVWDI~SGLRK
AN3026.1  	SDDQTIRIWNWQNRSLI1CTMTGHNHYVMCAQFHPTEDLIASASLDQSVRIWDI~SGLRK
          	****************: *********.*******.***:.******:**:*** *****

MG03511.1 	KHSAPTSSLSFEDQMARNNANQTDMFGNTDAVVKFVLEGHDRGVNWVAFHPTMPLIVSAG
NCU10066.1	KHSAPTS-MSFEDQVARANANQTDMFGNTDAVVKFVLEGHDRGVNWVAFHPTMPLIVSAG
FG07939.1 	KHSAPTS-MSFEDQMARANQNQTDMFGNTDAVVKFVLEGHDRGVNWVAFHPTMPLIVSAG
AN3026.1  	KHSAPTT-MSFEDQMARANNAQADMFGNTDAVVKFVLEGHDRGVNWVAFHPSLPLIVSAG
          	******: :*****:** *  *:****************************::*******

MG03511.1 	DDRLVKLWRMSE~TKA~WEVDTCRGHFQNASGCLFHPHQDLILSVGEDKTIRVWDLNKRT
NCU10066.1	DDRLIKLWRMSE~TKA0WEVDTCRGHFQNASGCLFHPHQDLILSAGEDKTIRVWDLNKRT
FG07939.1 	DDRLVKLWRMSE~TKA~WEVDTCRGHFQNASGCLFHPHQDLILSAGEDKTIRVWDLNKRT
AN3026.1  	DDRLIKLWRMSD1TKA~WEVDTCRGHFQNASACLFHPHQDLILSVGEDKTIRAWDLNKRT
          	****:******: *** **************.************.*******.*******

MG03511.1 	GVQSFKRENDRFWVIAAHPEINLFAAGHDNGVMVFKLERERPASAVNQNVLFYINKEKHV
NCU10066.1	VVNTFKRENDRIWVIAAHPEINLFAAGHDNGVMVFKLERERPASAVYQNNIFYITKEKHV
FG07939.1 	AVQSFKRENDRFWVIAAHPEINLFAAGHDNGVMVFKLERERPASAVHQNLLFYVTKEKHV
AN3026.1  	PVQSFKRDVDRFWVIAAHPEINLFAAGHDTGVMVFKLERERPASAVYQNQLFYITKEKHV
          	 *::***: **:*****************.**************** ** :**:.*****

MG03511.1 	RSYDFQKNIESPTLLSLKKVGPAWVPPRTLSYNPAERSVLVTSSADNGTYELISLPREGS
NCU10066.1	KMYDFQKNVESPTLLSLKKLGSPWVPPRTLSYNPAERSILVTSPADGGSYELITLPRDGS
FG07939.1 	RSYDFQKNIESPTLLSLKKLGSAWVAPRTLSFNPAERSILVTSPADGGSYELVNLPKDGS
AN3026.1  	KSFDFAKNVESPSMLSLRKLGSPWVPPRTLSYNPAERAVLVTSPADGGVYELINLPRDAT
          	: :** **:***::***:*:*..**.*****:*****::****.**.* ***:.**::.:

MG03511.1 	GAIEPTESKRGQGNSAIFVARNRFAVLNVASQTIDIKDLSNNTTRSFKPPHGTTDIYFGG
NCU10066.1	GAIEPTESKRGQGNSAVFVARNRFAVLNTATQTVDIKDLTNNTTRSFKPPVGTTDIYFGG
FG07939.1 	GAIEPAESKRGAGSSAIFVARNRFAVLNTANQTIDIKDLSNNTTRSFKPPTGTTDIYFGG
AN3026.1  	GAIEPTDVKRGQASSAVFVARNRFAVFSQANQQVDIKDLSNSTTKSIKPPAGTTDIYFGG
          	*****:: *** ..**:*********:. *.* :*****:*.**:*:*** *********

MG03511.1 	PGNLLIITPTAVHLYEIQQKKTIAELAVNGVKYVVWSNDGLYAALLSKHNVTIVTKNLEQ
NCU10066.1	TGNLLIITPTAVHLYDIQQRKSTAELAVTGVKYVVWSNDGLYAALLSKHNVTIVTKTLEQ
FG07939.1 	TGNLLIITPTAVHLYDIQQKKSTAELAVNGVKYVVWSNDGLYAALLSKHNVTIVSKTLEQ
AN3026.1  	TGSLLFITPTSVVLFDIQQKKQLAELAVSGVKYVVWSNDGLYAALLSKHNVTIVTKTLEQ
          	.*.**:****:* *::***:*  *****.*************************:*.***

MG03511.1 	VSTLHETIRIKSATWDDAGVLLYSTLNHVKYTLLNG~DNGIVRTLDQTVYLVRVKGRNAH
NCU10066.1	VSTLHETIRLKSAAWDDAGVLLYSTLNHVKYTLLNG~DNGIVCTLDQTVYLTRVKGRNVY
FG07939.1 	VSTLHETIRIKSATWDDAGVLLYSTLNHVKYTLLNG~DNGIVRTLDQTVYLVKVKGRNVY
AN3026.1  	VSSLHETIRIKSAAWDDAGVLLYSTLNHVKYSLLNG2DNGIIRTLDQTVYLVKVKGRSVY
          	**:******:***:*****************:**** ****: ********.:****..:

MG03511.1 	CLDRNAKPKILLIDPTEYRFKLSLVKRNYEEMLHIIKTSSLVGQSIISYLQKKGYPEIAL
NCU10066.1	CLDRAAKPKILQIDPTEYRFKLALVKRNYEEMLHIIQNSSLVGQSIIAYLQKKGYPEIAL
FG07939.1 	CLDRAAKPRILQIDPTEYRFKLALVKRNYEEMLHIIRNSSLVGQSIISYLQKKGYPEIAL
AN3026.1  	CLDRNAKPRTLEIDPTEYRFKLALVKRNYDEMLQIIKTSSLVGQSIISYLQKKGYPEIAL
          	**** ***: * **********:******:***:**:.*********:************

MG03511.1 	QFVQDPTTRFELAIECGNLEVAVEMAKQLDRPKLWTRLSTEALAHGNHQVVEMCYQKLKQ
NCU10066.1	QFVQDPTTRFELAIECGNLEVAVEMAKQLDKPKLWTRLSQEALAHGNHQIVEMCYQKLKH
FG07939.1 	QFVQDPTTRFDLAVECGNLDVAVEMAKELDKPKFWTRLGTEALAHGNHQVVEMCYQKLKQ
AN3026.1  	QFVQDPQTRFELALECGNLDVAIEMARELDRPNLWNRLGIEALAHGNHQIVEMAYQKQRN
          	****** ***:**:*****:**:***::**:*::*.**. *********:***.*** ::

MG03511.1 	FDKLSFLYLSTGDTTKLARMAKIAEHRGDFTARFQDALFLGEVEDRIQMFKEIDLY1PMA
NCU10066.1	FDKLSFLYLTTGDHDKLSRMAKIAEHRGDFTSRFQNALYLGEVQDRIQMFKEIDLY~PLA
FG07939.1 	FDKLSFLYLATGDRAKLARMAKIAEHRGDFTSRFQNAVYLGEVEDRIQMFKEIDLY1PLA
AN3026.1  	FDKLSFLYLSIGDQEKLSRMAKIAEHRGDFTSRFQNAIYRGDVEDRIQMFKEVDLY1PLA
          	*********: **  **:*************:***:*:: *:*:********:*** *:*

MG03511.1 	YMTAKSHGLDEECQAILEATGLTEDQLTMPKIGEALTPPKAVVPTHKANWPTRATSQSVF
NCU10066.1	YMTAKSHGLEDECQEILEATGLTEDQLSMPTLGEPLTPPKPVVPTYKANWPTKAASQSFF
FG07939.1 	YMTAKSHGLEEECQSILEATGVAEEDLTLPALGSPLSTTAPIVPTFKASWPTKATSQSFF
AN3026.1  	YLTAKAHGLTEEAESILEVVGLSEDQVSLPTIEAPPQIPQPIVATYKASWPVKAAAHSSF
          	*:***:*** :*.: ***..*::*:::::* :  .   . .:*.*.**.**.:*:::* *

MG03511.1 	EKALLGQMEGLSLDDESGAAANGY-AEEG---EEPSSKTGALVDVDDEEDAAGWDMGDDI
NCU10066.1	EKALLGEVEGLSLEDQPSAAPNGLGLEEGGADEPATKKAGALAEGDEDEDAAGWDMGDDI
FG07939.1 	EKALLGQVEGLSLEDEPAAANTGF--EDAMEIDSAAKRNGTLID-DDDEDVAGWDMGDDD
AN3026.1  	EKALLGEVS----AGDEEAAELGFEPEEE-----GAVTAGEALE-DEDEDAAGWDMGDEI
          	******::.     .:  **  *   *:       :   *   : *::**.*******: 

MG03511.1 	VPEVDSDFVNVESAETGGAGSSEADLWARNSPLAVDHVAGGSFESAMQLLNRQVGAVNFA
NCU10066.1	VPEVEEGFVNVESADAGGAASSEADLWSRNSPLAVDHVAGGSFESAMQLLNRQVGAVNFA
FG07939.1 	IPEADSDFVNVESVDAGGAASSEADMWARNSPLAVDHVAGGSFETAMQLLNRQVGAVDFA
AN3026.1  	NVEEDVDFVNVDSAEAG-AGSVEADLWARNSPLAADHVAAGSFDTAMQLLNRQVGAVHFA
          	  * : .****:*.::* *.* ***:*:******.****.***::************.**

MG03511.1 	PLKDRFLEVYKASKTYLPASVGLPALVNYVRRTTDETDPRKVLPIIPRDLESLATNDLQQ
NCU10066.1	PLKPRFLEVYQASKTYLPAAASLDPLVNYVRRNPEETDPRKVLPIIPRDLEYLASNDLQR
FG07939.1 	PLKSRFLEIYSASKTFLPASEGLPPLVNYVRRTLEETDPRKVLPIIPRDLEHLASNDLQA
AN3026.1  	PLKPRFLEIFKASKTYLSATPGLPPLVNYVRRTVDETDSRKVLPAIPRDLETIASVDLQE
          	*** ****::.****:*.*: .* .*******. :***.***** ****** :*: *** 

MG03511.1 	GYSTMKSNKLEEGVKVFRDILHAVLVNAVSSEGEVAEAKKLITSAAEYAVAMDIELARRK
NCU10066.1	GYDAMRANKLEDGVKIFKGILHSVLVNAVGSDGEVAEAKKLIASACEYSVAMSIELARRQ
FG07939.1 	GYDSMKTNKLEAGIGIFKGILHSILVNAVTSEDEVSEAKKLITSASEYAVAMSIELSRRQ
AN3026.1  	GYAAMRANKLEDGVKIFKNILYSVLVNVVSSEAEVEQAKKIIATSREYILAMTIELERRA
          	** :*::**** *: :*:.**:::***.* *: ** :***:*::: ** :** *** ** 

MG03511.1 	LGAPDAVAKNPELLKRSLELSAYFTIPKIEVPHRQLALLNAMNLAARSKNYNSALSFANR
NCU10066.1	LGSPDQVNSDPSLQKRSLELSAYFTIPKIEVPHRQLALLNAMNLAVRSKNYSSALSFANR
FG07939.1 	LGSPEVVAQDPEKLQRSLELSAYFTIPKIEVPHRQLALLSAMQLAIRNKNYNSALSFANR
AN3026.1  	LS-----TDTPEGLKRSLELSAYFTIPKLEVAHRQLALMAAMKLAFTHKNYSSALSFANR
          	*.      . *.  :*************:**.******: **:**   ***.********

MG03511.1 	ILANGGASKILET0AKKTKAQCERNPNDAVEIEFDQFAEFEVCAASHTPIYSGTAYEECA
NCU10066.1	ILANGGATKITES0AKRTKAQCERNPHDAVEIEFDQFAEFEICAASFTPIYSGTPYEECA
FG07939.1 	IIANGGATKIVEN0AKKTKAQCERNPNDAIEIEFDQFAEFDVCAASHTPIYSGTAYEECA
AN3026.1  	MLANGGSAKLLDQ0AKKVKAQCERNPQDSIEIEFDQFAEFDICAASHTPIYSGSPSVSDP
          	::****::*: :  **:.********:*::**********::****.******:.  . .

MG03511.1 	FDGSKYHTKYKGTVCAVCQVCEVGKHGSGLKL~FA---------
NCU10066.1	FDGAKYHTKYKGTVCTVCEVCEVGKHGSGLKL~FA---------
FG07939.1 	FDGSKYHTKYKGTVCRVCEVCEIGKHGSGLKL~FA---------
AN3026.1  	FTGAKYHEQYKGSVDRISEVTEIGAPASGLRL2YMDLLPVFYNS
          	* *:*** :***:*  :.:* *:*  .***:* : .  .   .:
```
